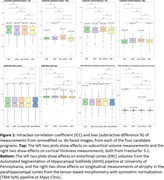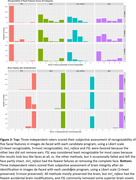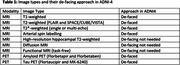# Implementation and Validation of Face De‐Identification (de‐facing) in ADNI‐4

**DOI:** 10.1002/alz.085266

**Published:** 2025-01-09

**Authors:** Christopher G. Schwarz, Duygu Tosun, Paul A. Yushkevich, Mark Choe, Stephanie Rossi, Sandhitsu R. Das, Ranjit Ittyerah, Evan Fletcher, Pauline Maillard, Baljeet Singh, Danielle J. Harvey, Ian B. Malone, Lloyd Prosser, Matthew L. Senjem, Leonard C. Matoush, Chadwick P. Ward, Carl M. Prakaashana, Charles Decarli, Michael S. W. Weiner, Clifford R. Jack, The Alzheimer's Disease Neuroimaging Initiative

**Affiliations:** ^1^ Mayo Clinic, Rochester, MN USA; ^2^ University of California, San Francisco, San Francisco, CA USA; ^3^ San Francisco Veterans Affairs Medical Center, San Francisco, CA USA; ^4^ University of Pennsylvania, Philadelphia, PA USA; ^5^ University of California, Davis, Davis, CA USA; ^6^ Alzheimer's Disease Research Center, University of California Davis, Sacramento, CA USA; ^7^ University of California, Davis School of Medicine, Sacramento, CA USA; ^8^ Dementia Research Centre, UCL Queen Square Institute of Neurology, London UK; ^9^ University of California Davis, Davis, CA USA

## Abstract

**Background:**

Recent advances in automatic face recognition have increased the risk that de‐identified research imaging data could be re‐identified from face imagery in brain scans.

**Method:**

An ADNI committee of independent imaging experts evaluated 11 published techniques for face‐deidentification (“de‐facing”) and selected four algorithms (FSL‐UK Biobank, HCP/XNAT, mri_reface, and BIC) for formal testing using 183 longitudinal scans of 61 racially and ethnically diverse ADNI participants, evaluated by their facial feature removal on 3D rendered surfaces (confirming sufficient privacy protection) and by comparing measurements from ADNI routine image analyses on unmodified vs. de‐faced images (confirming negligible side effects on analyses).

**Result:**

Effects of the de‐facing methods on FreeSurfer at UCSF (Figure 1‐top), ASHS at University of Pennsylvania (Figure 1 bottom‐left), and longitudinal atrophy at Mayo Clinic (Figure 1 bottom‐right) were comparable with each other and sufficiently small vs. unmodified scans to recommend de‐facing in ADNI‐4. The FSL and mri_reface methods were more thorough in removing facial features, partly because these also removed ears (Figure 2‐top). mri_reface was recommended for ADNI‐4 primarily because fewer (zero) images had modified brain (Figure 2‐bottom), and ADNI leadership approved de‐facing with mri_reface. ADNI performed a second validation of its latest version on another diverse sample of 100 ADNI‐3 participants. These analyses included: boundary shift interval at University College London, where average differences in measurements were all <0.05%; cortical white matter hyperintensities at UC Davis, where they found “minimum impact” (R>0.97); and others.

**Conclusion:**

ADNI‐4 de‐faces all indicated image types (Table 1) before subsequent pre‐processing, analyses, and public release. Sites upload images to the central repository (LONI), where they are queued to the MRI Core at Mayo Clinic for de‐facing. Image types requiring de‐facing are de‐faced using mri_reface. Trained analysts inspect de‐faced images to confirm complete removal of the face and complete non‐modification of the brain, and any flagged images are re‐processed manually until issues are resolved. De‐faced images are converted back to DICOM and re‐uploaded for further distribution. Image types not requiring de‐facing are indicated to proceed directly to subsequent steps. In summary, ADNI‐4 has adopted mri_reface because it successfully de‐faces without substantially affecting brain measurements.